# Genetic and Epigenetic Studies in Diabetic Kidney Disease

**DOI:** 10.3389/fgene.2019.00507

**Published:** 2019-06-07

**Authors:** Harvest F. Gu

**Affiliations:** Center for Pathophysiology, School of Basic Medicine and Clinical Pharmacy, China Pharmaceutical University, Nanjing, China

**Keywords:** diabetic kidney disease, diabetes, end-stage renal disease, genetics, epigenetics, phenotypes

## Abstract

Chronic kidney disease is a worldwide health crisis, while diabetic kidney disease (DKD) has become the leading cause of end-stage renal disease (ESRD). DKD is a microvascular complication and occurs in 30–40% of diabetes patients. Epidemiological investigations and clinical observations on the familial clustering and heritability in DKD have highlighted an underlying genetic susceptibility. Furthermore, DKD is a progressive and long-term diabetic complication, in which epigenetic effects and environmental factors interact with an individual’s genetic background. In recent years, researchers have undertaken genetic and epigenetic studies of DKD in order to better understand its molecular mechanisms. In this review, clinical material, research approaches and experimental designs that have been used for genetic and epigenetic studies of DKD are described. Current information from genetic and epigenetic studies of DKD and ESRD in patients with diabetes, including the approaches of genome-wide association study (GWAS) or epigenome-wide association study (EWAS) and candidate gene association analyses, are summarized. Further investigation of molecular defects in DKD with new approaches such as next generation sequencing analysis and phenome-wide association study (PheWAS) is also discussed.

## Introduction

Diabetes is a major public health problem that is approaching epidemic proportions globally. According to the latest report from the IDF, the prevalence of diabetes will increase from 425 million persons in 2017 to 629 million by 2045 (IDF 2017^1^). Diabetic kidney disease (DKD, previously termed diabetic nephropathy, DN) is a microvascular complication and progresses gradually over many years in approximately 30–40% of individuals with T1D and T2D mellitus (Harjutsalo and Groop, 2014; Thomas et al., 2015; Barrett et al., 2017). DKD is now the main cause of chronic kidney disease (CKD) worldwide and the leading cause of end-stage-renal disease (ESRD) requiring renal replacement therapy (dialysis or transplantation). The presence of CKD is the single strongest predictor of mortality for persons with diabetes (Dousdampanis et al., 2016; Papadopoulou-Marketou et al., 2017). Pathological findings in DKD include glomerular hypertrophy, mesangial matrix expansion, reduced podocyte number, glomerulosclerosis, tubular atrophy and tubulointerstitial fibrosis. Clinical criteria used to diagnose the subjects with DKD are urine ACR higher than 300 mg/g, while microalbuminuria is diagnosed when ACR is between 30–300 mg/g (Bouhairie and McGill, 2016). Accumulating evidence has indicated that podocyte loss and epithelial dysfunction play important roles in DKD pathogenesis with further progression associated with inflammation but the exact molecular mechanisms responsible for DKD are not fully known (Badal and Danesh, 2014; Reidy et al., 2014; Gnudi et al., 2016).

**FIGURE 1:**
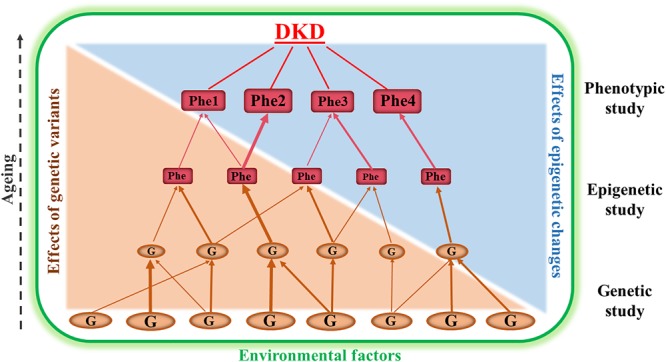
This is a schematic diagram representing the relationship between genetic, epigenetic and phenotypic studies in diabetic kidney disease (DKD). Genetic association studies are fundamentally important for identification of susceptibility or resistance genes (G). Epigenetic studies analyzing genomic DNA methylation changes, chromosome histone modification and ncRNA regulation are useful for dissecting the interaction of the genes with environmental factors. The combined data from genetic, epigenetic and phenotypic (Phe) studies may provide the opportunity for us to understand new pathways underlying the pathogenesis of DKD and to discover new biomarkers for early diagnosis and to find targets for prevention and treatment programs of this disease. The different sizes of the ‘G” and “Phe” represent the variation of genetic and phenotypic effects.

Both clinical and epidemiological studies have demonstrated that there is familial aggregation of DKD in different ethnic groups, indicating that genetic factors contribute to development of the disease. Furthermore, genetic risk factors in DKD interact with the environmental factors (for example, lifestyle, diet and medication) (Freedman et al., 2007a; Murea et al., 2012; Thomas et al., 2012; Kato and Natarajan, 2014). Figure 1 is a schematic diagram representing the relationship between genetic, epigenetic and environmental factors that are involved in the development and progression of DKD. Genetic studies of DKD are mainly focused on association analyses between genomic DNA variation (for example, single nucleotide polymorphisms, SNPs, copy number variants, CNVs, and microsatellites) and clinical phenotypes of the disease (Freedman et al., 2007a; Gu and Brismar, 2012; Thomas et al., 2012; Florez, 2016). Epigenetics studies of DKD examine potentially heritable changes in gene expression that occur without variation in the original DNA nucleotide sequence (Villeneuve and Natarajan, 2010; Kato and Natarajan, 2014; Thomas, 2016; Keating et al., 2018). Therefore, epigenetic studies of DKD may provide information to help understand how environmental factors modify the expression of genes that are involved in DKD progression. Combined genetic, epigenetic and phenotypic studies together may generate information to understand new pathogenic pathways and to search for new biomarkers for early diagnosis and prediction as part of prevention programs in DKD. The results may also be useful in finding novel targets for the treatment of DKD.

SNPs are the most common form of genomic DNA variation. The updated dbSNP database of more than 500 million reference SNPs (rs) with allele frequency data^2^ has provided fundamental information for genetic studies of complex diseases including, DKD. The genetic studies in DKD have implicated previously unsuspected biological pathways and subsequently improved our knowledge for understanding of the genetic basis of the disease. For most common traits studied in DKD, however, the identified genes and their SNPs only explain a fraction of associated risk, suggesting that human genomic DNA variations are only a part of underlying susceptibility to DKD. This has led to evolving interest in epigenetics to help explain some of the missing heritability of DKD. Epigenetic mechanisms mainly consist of DNA methylation, chromosome histone modification and non-coding RNA (ncRNA) regulation (Kato and Natarajan, 2014; Allis and Jenuwein, 2016). Epigenetic related ncRNAs include miRNA, siRNA, piRNA, and lncRNA (Holoch and Moazed, 2015). There are more than 30,000 identified CpG islands in the human genome. Detailed information for these CpG islands can be found in the public database^3^. The CpG islands are defined as stretches of DNA > 200 bp long with a GC percentage greater than 50% and an observed-to-expected CpG ratio of more than 60%. The CpG islands are often found at promoters and contain the 5′ end of the transcript, while DNA methylation occurs at 5′-cytosines of “CpG” dinucleotides^4^ (Cross and Bird, 1995). In DKD, the effects of DNA methylation have been studied in terms of transgenerational inheritance of the disease to explore environmental and other non-genetic factors that may influence epigenetic modifications in the genes involved in DKD (Deaton and Bird, 2011; Jones, 2012). Identification of differentially methylated CpG sites in promoters or other functional regions of genes and the analysis of the DNA methylation changes that are associated with DKD have become the most common approaches used in epigenetic studies of the disease (Villeneuve and Natarajan, 2010; Kato and Natarajan, 2014; Thomas, 2016). Furthermore, ncRNAs, particularly long ncRNAs are known to be involved in epigenetic processes. ncRNAs certainly play an important role in chromatin formation, histone modification, DNA methylation and consequently gene transcription silencing.

Genetic and epigenetic studies of DKD, initially using candidate gene approaches and more recently at genome-wide scale (known as GWAS and EWAS), have been undertaken to identify many genes conferring susceptibility or resistance to DKD. In this review, clinical phenotypes, research approaches and experimental designs that have been used for genetic and epigenetic studies of DKD are described. These research approaches and experimental designs can also be used for study of CKD. Current information from genetic and epigenetic studies of DKD is summarized. Further investigation of molecular defects in DKD with new generation sequencing analyses and phenome-wide association studies (PheWAS) are discussed.

## Biological Material, Research Approaches and Study Designs Used in Genetic and Epigenetic Investigations of Diabetic Kidney Disease

Two major research approaches either at genome-wide scale or focused on candidate gene(s) have been widely used for comparative studies between cases (patients with DKD) and controls (diabetes patients without DKD). Case-control studies by recruiting large numbers of subjects can increase the statistical power of reported associations. The aim is to discover the genes presented differentially in genomic structure or genetic expression. Genome-wide or epigenome-wide association studies (GWAS or EWAS) are hypothesis-generating approaches (Rakyan et al., 2011; Do et al., 2017; Lappalainen and Greally, 2017). These study designs have benefited from rapid development of human genome research, including the creation of publicly available databases of SNPs, haplotypes and CpG islands and the rapid technical improvements in analyzing genomic variation using high-throughput techniques and high-density SNP or CpG arrays. Another approach is to focus on candidate genes and study a more limited number of genes potentially involved in the pathogenesis of DKD based upon our known knowledge or hypothesis. In genetic and epigenetic studies of DKD, DNA samples used are commonly extracted from peripheral blood samples because they are clinically accessible. Dick et al. (2014) have comparatively analyzed DNA methylation changes related to BMI by using both approaches of whole-blood DNA methylation profiling and adipose tissue specific methylation measurement. Data suggests that analysis of blood DNA methylation is worthwhile because the results can reflect the DNA methylation changes in relevant tissues for a particular phenotype. Nevertheless, there is still limited information concerning the correlation between whole blood DNA methylation profiles and kidney tissue specific DNA methylation changes in part due to the heterogeneity of cell types within the kidney. To improve the tissue specific DNA methylation analysis of kidney diseases, including DKD, it is necessary to construct biobanks of renal biopsies. Karolinska Institutet has established a biobank in KaroKidney with more than 750 renal biopsies^5^. The advantages and limitations of these two approaches, as well as the clinical materials and experimental design used in genetic and epigenetic studies of DKD are summarized in Table 1.

**Table 1:** Clinical material, research approaches and experimental designs used in genetic and epigenetic studies of diabetic kidney disease.

	Study	Advantage	Disadvantage
Clinical material	Blood or saliva	Clinical accessible	Possible bias from mixed cell types
	Kidney tissues	Gene specific methylation and expression can be analyzed	Difficult to access
	Renal cell lines	Intervention and mechanism study	*In vitro* experiment
Research approach	Candidate gene DNA variation or methylation analysis	Study of candidate genes with potential biological functions	Less information on the studied genes
	Global genomic DNA variation or methylation analyses	General information of DNA polymorphisms and methylation in genome wide scale	Analysis of repeated sequence alteration and methylation changes Lack of gene specific information
	Genome or epigenome-wide association studies	Numerous SNP, CNV or CpG sites methylation information in genome wide scale	Higher cost Strict validation is needed
Experimental design	Case-control study	Many cohorts exist	Difficult to control genetic and environmental confounders
	Twin study	Control for genetics	Few large cohorts
	Family study	Study of potential inheritance	Few large cohorts
	Longitudinal study	Determine causality	Time consuming

*CNV, copy-number variation; CpG sites, the regions of DNA where a cytosine nucleotide is followed by a guanine nucleotide in the linear sequence of bases along its 5′ → 3′ direction; SNP, single-nucleotide polymorphism.*

## Recent Data From Genetic Studies in Diabetic Kidney Disease

Considerable amounts of data from genetic studies in DKD have accumulated. A list of the genes that are reported to be associated with susceptibility or resistance to DKD are summarized in Table 2. The genes are listed in alphabetical order. Surprisingly, there are more than 150 genes. Most of them have been identified by genetic association studies employing candidate gene approaches over the past 20 years. Furthermore, a number of GWAS in DKD have been published in the last 10 years. By using GWAS approaches, approximately 33 genes have been found to be associated with the DKD, i.e., *ABCG2, AFF3, AGER, APOL1, AUH, CARS, CERS2, CDCA7/SP3, CHN2, CNDP1, ELMO1, ERBB4, FRMD3, GCKR, GLRA3, KNG1, LIMK2, MMP9, NMUR2, MSRB3/HMGA2, MYH9, PVT1, RAET1L, RGMA/MCTP2, RPS12, SASH1, SCAF8/CNKSR3, SHROOM3, SLC12A3, SORBS1, TMPO, UMOD*, and *ZMIZ1* (Hanson et al., 2007; Sandholm et al., 2012, 2014; Maeda et al., 2013; Thameem et al., 2013; Bailey et al., 2014; Palmer et al., 2014; Guan et al., 2016; Teumer et al., 2016; Lim et al., 2017; Roden, 2017; Charmet et al., 2018; van Zuydam et al., 2018). However, most of these genes (∼80%) reportedly associated with DKD still need to be confirmed by further replication studies and detailed analysis of their functional role in DKD in experimental models. Polymorphisms in these candidate genes association with DKD studies are listed in Table 2A, while their potential biological relevance and genetic effects in DKD are briefly described. Of them, 34 genes are originally predicted by GWAS and the statistical association with DKD summarized in Table 2B.

**Table 2A:** Current data from genetic association studies in diabetic kidney disease by using candidate gene approach.

Gene symbol	Genomic DNA polymorphisms	Disease
*ABCG2*	rs2231142	T2D-uric acid
*ACACB*	rs2268388	T2D-DKD
*ACE*	rs4646994 (289bp Alu I/D), rs4343, rs1799752, rs1800764, rs12449782	T1D-DKD, T2D-DKD, T2D-ESRD
*ADPOQ*	rs266729, rs17300539, rs2241766, rs1063537, rs2241767, rs2082940	T1D-DKD, T2D-DKD
*ADRB2*	Arg16Gly, Gln27Glu	T2D-eGFR
*AFF3*	rs7583877	T1D-ESRD
*AGER*	rs2070600, rs2071288	T2D-DKD
*AGT*	rs5050, rs4762, Met235Thr	T2D-DKD
*AGTR1*	rs5186, +1166A/C, -106C/T, rs12695897	T1D-DKD, T2D-ESRD
*AGTR2*	+1675G/A, +1818A/T	T1D-DKD
*AKR1B1*	rs759853	T2D-DKD, T2D-ESRD
*ALOX12*	rs14309	T2D-DKD+CVD
*APOE*	e4 allele, e2/e3 alleles	T2D-DKD
*APOL1*	rs136161, rs713753, rs767855, Ser342Gly, Ile384Met	T2D-ESRD
*AUH*	rs773506	T2D-ESRD
*BID*	rs181390	T1D-ESRD
*CALD1*	rs3807337	T1D-DKD
*CARS*	rs452041, rs739401	T1D-DKD, T2D-DKD
*CASR*	rs3804594	T2D-DKD
*CAT*	rs1001179	T2D-ESRD
*CERS2*	rs267734, rs267738	T1D-DKD, T2D-DKD
*CDH13*	rs11646213, rs3865188	T1D-ESRD
*CFH*	rs379489	T2D-ESRD
*CHN2*	rs39059	T1D-DKD
*CNDP1*	(CTG)5, rs4892249, rs6566815, rs2346061, rs1295330, rs6566810, rs11151964, rs17817077	T2D-dialysis, T2D-DKD, T1D-ESRD, T2D-ESRD
*CNDP2*	rs7577, rs4892247	T2D-ESRD
*CYP11B2*	-344T/C	T2D-DKD
*COQ5*	rs1167726, rs614226, rs1167725	T1D-ESRD
*COX6A1*	rs12310837	T1D-ESRD
*COX10*	rs7213412	T1D-ESRD
*CUBN*	rs1801239	T1D-albuminuria, T2D-ESRD
*CYBA*	rs4673, rs9932581	T1D-ESRD, T2D-DKD
*eNOS*	-786C/T, +786T/C, +894G/T, Glu298Asp	T1D-DKD, T2D-DKD
*ELMO1*	rs741301, rs1345365, rs11769038, rs10951509, rs1882080, rs6462776, rs6462777	T1D-DKD, T1D-ESRD, T2D-DKD
*ENPP1*	rs1044498, rs7754586, rs1974201	T1D-DKD, T2D-DKD, T2D-ESRD
*EPHX2*	rs751141	T2D-DKD
*EPO*	rs1617640	T1D-ESRD, T2D-DKD
*ERBB4*	rs7588550	T1D-DKD
*ESR1*	rs12197043, rs11964281, rs1569788, rs9340969	T2D-DKD
*FNDC5*	rs16835198	T2D-DKD
*FRMD3*	rs1888747, rs10868025, rs942280, rs942278, rs942263, rs1535753, rs2378658, rs13288659	T1D-ESRD, T2D-DKD
*GAS6*	Intron 8, c.834+7G/A	T2D-DKD
*GATC*	rs2235222, rs7137953	T1D-ESRD
*GCK*	rs730947	T2D-ESRD
*GCKR*	rs1260326	T2D-eGFR
*GFPT2*	Ile147Val	T2D-DKD
*GLRA3*	rs1564939	T1D-AER
*GPX1*	rs3448	T1D-DKD
*GREM1*	rs1129456	T1D-DKD
*GSTP1*	rs1695 (Ile105Val)	T2D-DKD, T2D-ESRD
*H19-IGF2 cluster*	rs2839698, rs10732516, rs201858505	T2D-DKD
*HIF1α*	rs11549465 (Pro582Ser)	T1D-DKD, T2D-DKD
*HO1*	-413T/A	T2D-DKD
*HSP70*	rs2763979, rs2227956	T2D-DKD
*ICAM1*	rs5498	T1D-DKD, T2D-DKD
*IGFBP1*	rs1065780, rs3828998, rs3793344, rs4619	T2D-DKD
*IGF2BP2*	rs4402960	T2D-DKD
*IL1α*	-889C/T	T2D-DKD
*IL1β*	rs16944, -511C/T	T2D-DKD
*IL6*	-634G/C, -174G/C, rs1800796, rs1524107, rs1800795, rs1800796	T2D-DKD
*IL10*	-819T/C, -592A/C, -1082A/G	T2D-DKD
*IL18*	rs360719	T2D-DKD
*INSR*	rs2059806	T2D-DKD
*IRAK4*	rs4251532	T2D-DKD
*KCNQ1*	I/D in intron 12, rs2237897	T2D-eGFR, T2D-DKD
*KLRA1*	rs2168749	T1D-ESRD
*KNG1*	+7965C/T	T1D-DKD
*LIMK2*	rs2106294	T2D-ESRD
*LTA*	Thr60Asn	T1D-DKD
*LRP2*	rs17848169	T2D-ESRD
*MAPRE1P2*	rs1670754	T1D-ESRD
*MCF2L2*	Leu359Ile	T1D-DKD
*MGP*	-138T/C	T2D-DKD
*MME*	rs3796268, rs3773885	T1D-DKD
*MMP12*	rs1277718, rs652438, Asn357Ser	T1D-DKD
*MMP9*	(CA)n in promoter, rs481480, rs2032487, rs4281481, rs3752462, rs3918242	T2D-ESRD, T2D-DKD
*NMUR2*	rs982715, rs4958531, rs4958532, rs4958535	T1D-DKD
*MSC*	rs9298190	T1D-ESRD
*MT2A*	rs28366003	T2D-DKD
*MTHFR*	rs1801133	T1D-DKD, T2D-DKD
*MTOR*	rs7212142	T2D-DKD
*MyD88*	rs6853	T2D-DKD
*MYH9*	rs5750250	T2D-ESRD
*NCALD*	rs1131863, +999T/A, +1298A/C, +1307A/G	T2D-DKD
*near IRS2*	rs1411766	T1D-DKD, T1D-ESRD, T2D-DKD
*NOS2*	rs1137933	T2D-DKD
*NOS3*	rs3918188, Glu298Asp, Gly894Thr	T1D-DKD, T2D-DKD
*NQO1*	rs1800566	T2D-DKD
*NPHS1*	rs35238405	T2D-ESRD
*NPY*	Leu7Pro	T1D-DKD
*PACRG*	rs2147653, rs1408705	T1D-ESRD
*PAI1*	4G/5G	T2D-DKD
*PARK2*	rs4897081	T2D-DKD
*PARP1*	C410T, G1672A, Val762Ala	T2D-DKD
*PFKFB2*	rs17258746, rs11120137	T2D-DKD
*PLEKHH2*	rs1368086, rs725238, rs11886047	T1D-DKD
*PLXDC2*	rs1571942, rs12219125	T1D-DKD
*PON1*	Leu55Met, Gln192Arg	T1D-DKD, T2D-ACR
*PON2*	rs12704795	T2D-DKD
*PPARG*	rs1805192, rs1801282	T1D-DKD, T2D-DKD
*PPARG2*	Pro12Ala	T2D-eGFR, T2D-DKD
*PPARGC1A*	Gly482Ser	T2D-DKD
*PRKAA2*	rs2746342, rs10789038	T2D-DKD
*PROX1*	rs340841	T2D-DKD
*PSMD9*	rs1043307, rs14259, +460A/G, +437T/C, Glu197Gly	T2D-DKD
*PRKCB1*	-1504C/T, -546C/T, -348A/G, -278C/T, -238C/G	T1D-DKD, T2D-eGFR
*PTX3*	rs2305619, rs2120243	T2D-DKD
*PVT1*	rs2648875, rs2720709	T2D-ESRD
*RAGE*	-429T/C, -374T/A, +2184A/G	T1D-ESRD, T2D-DKD
*RAET1L*	rs1543547	T1D-DKD
*RBP4*	rs3758538, rs10882278, rs7094671, rs12766992	T2D-eGFR
*REN*	rs41317140	T2D-DKD
*RREB1*	rs9379084, rs41302867	T2D-ESRD
*TOP1MT*	rs7387720, 724037	T1D-ESRD
*TXNRD2*	rs17745445, rs17745433, rs5992495, rs5992493	T1D-ESRD
*RPS12*	rs7769051	T2D-ESRD
*RTN1*	rs1952034, rs12431381, rs12434215	T2D-ESRD
*SASH1*	rs6930576	T2D-ESRD
*SCAF8/CNKSR3*	rs12523833	T2D-DKD
*SEMA6D/SLC24A5*	rs12917114	T1D-ESRD
*SERPINB7*	rs1720843	T2D-DKD
*SERPINE1*	4G/5G polymorphism	T2D-DKD
*SHROOM3*	rs1739721	T2D-eGFR
*SIK1*	rs2838302	T1D-ESRD
*SIRT1*	rs4746720	T2D-DKD
*SLC2A1*	rs3820589, HaeIII polymorphism	T1D-DKD, T2D-DKD
*SLC2A2*	+16459C/T	T1D-DKD
*SLC2A9*	rs11722228, rs3775948	T2D-uric acid
*SLC12A3*	rs11643718	T2D-DKD, T2D-ESRD
*SOD1*	rs2234694	T1D-DKD
*SOD2*	Ala9Val, Val16Ala	T1D-DKD
*SORBS1*	rs1326934	T1D-DKD
*SOX2*	rs11915160	T1D-DKD
*SPTLC2*	rs176903	T1D-ESRD
*SUMO4*	rs237025	T2D-DKD
*SUV39H2*	rs17353856	T1D-DKD
*TCF7L2*	rs7903146	T2D-DKD
*TGFβ1*	rs1800470	T1D-DKD, T2D-DKD
*THP*	rs12444268	T1D-DKD
*TMPO*	rs4762495	T1D-ESRD
*TNFα*	rs1800629, rs1800470, rs1800469, rs1800630, rs1799964	T2D-DKD, T2D-ESRD
*TRAF6*	rs16928973	T2D-DKD
*TRIB3*	rs2295490	T2D-DKD
*UMOD*	rs12917707, rs13333226	T2D-DKD
*VDR*	Raql variant	T2D-DKD
*VEGF*	-1499C/T, rs2010963	T1D-DKD, T2D-DKD
*VEGFA*	rs3025021	T1D-DKD
*WNT4/ZBTB40*	rs12137135	T1D-ESRD
*ZMIZ1*	rs1749824	T1D-ESRD
*miRNA-146a*	rs2910164	T1D-DKD, T2D-DKD
*miRNA-125*	rs12976445	T2D-DKD

**Table 2B:** Current data from genetic association studies in diabetic kidney disease by using genome wide association approach.

Gene symbol	Genomic DNA polymorphisms	*P*-value	Disease	References
*ABCG8*	rs4148217	*P* = 0.003	T2D-ESRD	Nicolas et al., 2015
*AFF3*	rs7583877, rs7562121	*P* = 1.2 × 10(-8) and <1 × 10(-6)	T1D-ESRD	Sandholm et al., 2012, 2017
*AGER*	rs2070600, rs2071288	*P* < 0.001	T2D-DKD	Lim et al., 2017
*AGTR1*	rs12695897	*P = 0.032*	T2D-ESRD	Palmer et al., 2014
*APOL1*	rs136161, rs713753, rs767855	*P* = 0.006–0.037	T2D-ESRD	Palmer et al., 2014
*AUH*	rs7735506	*P* = 2.57 × 10(-4)	T2D-ESRD	McDonough et al., 2011
*BID*	rs181390	*P* = 0.006	T1D-ESRD	Craig et al., 2009
*CARS*	rs452041, rs739401	*P* = 3.1 × 10(-6)	T1D-DKD, T2D-DKD	Pezzolesi et al., 2009b
*CERS2*	rs267734, rs267738	*P* = 0.0013 and 0.0015	T1D-DKD, T2D-DKD	Shiffman et al., 2014
*CDCA7-SP3*	rs4972593	*P* = 5 × 10(-8)	T1D-ESRD in women	Sandholm et al., 2013
*CHN2*	rs17157914	*P* = 0.029	T2D-ESRD	Palmer et al., 2014
*CNDP1*	rs4892249, rs6566815	*P* = 0.0043 and 0.0076	T2D-ESRD	Palmer et al., 2014
*CNTNAP2*	rs1989248	*P <* 1 × 10(-6)	T1D-ESRD	Sandholm et al., 2017
*ELMO1*	rs741301 rs1345365, rs11769038, rs10951509, rs1882080, rs6462776, rs6462777	*P* = 0.004	T2D-DKD	Wu et al., 2013
*ERBB4*	rs7588550	*P* = 2.1 × 10(-7)	T1D-DKD	Sandholm et al., 2012
*FRMD3*	rs942278, rs1888747, rs10868025, rs942280, rs942263, rs1535753, rs2378658, rs13288659	*P* = 5.0 × 10(-7)	T1D-ESRD, T2D-ESRD	Pezzolesi et al., 2009a; Freedman et al., 2011
*GABRR1*	rs9942471	*P* = 4.5 × 10(-8)	T2D-DKD	van Zuydam NR
*GCKR*	rs1260326	*P* = 3.23 × 10(-3)	T2D-eGFR	Deshmukh et al., 2013
*GLRA3*	rs1564939	*P* = 0.0013	T1D-AER	Sandholm et al., 2018
KLKB	rs4253311	*P* = 5.5 × 10(-8)	Plasma renin activity	Lieb et al., 2015
*KNG1*	rs5030062	*P* = 0.001	Plasma renin activity	Lieb et al., 2015
*LIMK2*	rs2106294, rs4820043	*P* = 7.49E-04 and 0.001	T2D-ESRD	McDonough et al., 2011
*MMP9*	rs481480, rs2032487, rs4281481	*P* = 0.038, 0.045 and 0.048 *P* = 0.053, 0.054 and 0.055	T2D-ESRD T2D-DKD	Freedman et al., 2009; Cooke et al., 2012
*MYH9*	rs5750250, rs92280	*P* = 4.3 × E(-4) *P* = 3 × 10(-7)	T2D-ESRD	Freedman et al., 2011; McDonough et al., 2011
*PTPN13*	rs61277444	*P* < 1 × 10(-6)	T1D-DKD	Sandholm et al., 2017
*PVT1*	rs2648875, rs2720709	*P* = 1.8–2.1 × (-7)	T2D-ESRD	Hanson et al., 2007
*RAET1L*	rs1543547	*P* = 1 × 10(-5)	T1D-DKD	McKnight et al., 2009
*RGMA-MCTP2*	rs12437854	*P* = 2 × 10(-9)	T1D-ESRD	Sandholm et al., 2012
*RPS12*	rs9493454	*P* = 8.79 × 10(-4)	T2D-ESRD	McDonough et al., 2011
*SHROOM3*	rs1739721	*P* = 3.18 × 10(-3)	T2D-eGFR	Deshmukh et al., 2013
*SLC12A3*	rs11643718	*P* = 0.021	T2D-DKD, T2D-ESRD	Tanaka et al., 2003
*TMPO*	rs4762495	*P* = 0.0006	T1D-ESRD	Craig et al., 2009
*UMOD*	rs12917707	*P* = 8.84 × 10(-4)	T2D-eGFR	Deshmukh et al., 2013
*ZMIZ1*	rs1749824	*P* = 8.1 × 10(-5)	T1D-ESRD	Craig et al., 2009

*Data were extracted from more than 300 references in PubMed and most studies were carryout with genetic association study of candidate gene(s). CNVs, Copy Number Variants; DKD, Diabetic Kidney Disease; eGFR, *estimated Glomerular Filtration Rate*; T1D, Type 1 Diabetes Mellitus; T2D, Type 2 Diabetes Mellitus; *ABCG*, ATP Binding Cassette Subfamily G; *ACACB*, Acetyl-CoA Carboxylase Beta; *ACE*, Angiotensin I Converting Enzyme; *ADPOQ*, Adiponectin; *ADRB2*, Adrenoceptor Beta 2; *AFF3*, AF4/FMR2 Family Member 3; *AGER*, Advanced Glycosylation End-Product Specific Receptor; *AGT*, Angiotensinogen; *AGTR*, Angiotensin II Receptor; *AKR1B1*, Aldo-Keto Reductase Family 1 Member B; *ALOX12*, Arachidonate 12-Lipoxygenase, 12S Type; *ApoE*, Apolipoprotein E; *APOL1*, Apolipoprotein L1; *AUH*, AU RNA Binding Methylglutaconyl-CoA Hydratase; *BID*, BH3 Interacting Domain Death Agonist; *CALD1*, Caldesmon 1; *CaSR*, Calcium-Sensing Receptor; *CARS*, Cysteinyl-TRNA Synthetase; *CAT*, Catalase; *CERS2*, Ceramide Synthase 2; *CDCA7*, Cell Division Cycle Associated 7; *CDH13*, Cadherin 13; *CHN2*, Chimerin 2; *CNDP*, Carnosine Dipeptidase; *COQ5*, Coenzyme Q5, Methyltransferase; *COX6A1*, Cytochrome C Oxidase Subunit 6A1; *COX10*, COX10, Heme A:Farnesyltransferase Cytochrome C Oxidase Assembly Factor; *CUBN*, Cubilin; CYBA, Cytochrome B-245 Alpha Chain; *CYP11B2*, Cytochrome P450 Family 11 Subfamily B Member 2; *ELMO1*, Engulfment And Cell Motility 1; *eNOS*, Nitric Oxide Synthase; *ENPP1*, Ectonucleotide Pyrophosphatase/Phosphodiesterase 1; *EPO*, Erythropoietin; *EPHX2*, Epoxide Hydrolase 2; *ERBB4*, Erb-B2 Receptor Tyrosine Kinase 4; *ESR1*, Estrogen Receptor 1; *FRMD3*, FERM Domain Containing 3; *FNDC5*, Fibronectin Type III Domain Containing 5; *GAS6*, Growth Arrest Specific 6; *GATC*, Glutamyl-TRNA Amidotransferase Subunit C; *GCK*, Glucokinase; *GCKR*, Glucokinase Regulator; *GFPT2*, Glutamine-Fructose-6-Phosphate Transaminase 2; *GLRA3*, Glycine Receptor Alpha 3; *GPX1*, Glutathione Peroxidase 1; *GREM1*, Gremlin 1, DAN Family BMP Antagonist; *GSTP1*, Glutathione *S*-Transferase Pi 1; *HIF1α*, Hypoxia Inducible Factor 1 Subunit Alpha; *H19*, H19, Imprinted Maternally Expressed Transcript; *HMGA2*, High Mobility Group AT-Hook 2; *HO1*, Heme Oxygenase 1; *HSP70*, Heat Shock Protein 70; *ICAM1*, Intercellular Adhesion Molecule 1; *IGF2*, Insulin Like Growth Factor 2; *IGFBP1*, Insulin Like Growth Factor Binding Protein 1; *IL*, Interleukin; *IRAK4*, Interleukin 1 Receptor Associated Kinase 4; *INSR*, Insulin Receptor; IRS2, Insulin Receptor Substrate 2; *KCNQ1*, Potassium Voltage-Gated Channel Subfamily Q Member 1; *KLRA1*, Killer Cell Lectin Like Receptor A1; *KNG1*, Kininogen 1; *LTA*, Lymphotoxin Alpha; *LIMK2*, LIM Domain Kinase 2; *MAPRE1P2*, MAPRE1 Pseudogene 2; *MCF2L2*, MCF.2 Cell Line Derived Transforming Sequence-Like 2; *MGP*, Matrix Gla Protein; *MME*, Membrane Metalloendopeptidase; *MMP*, Matrix Metallopeptidase; *MSC*, Musculin; *MTHFR*, Methylenetetrahydrofolate Reductase; *MT2A*, Metallothionein 2A; *MSRB3*, Methionine Sulfoxide Reductase B3; *MTOR*, Mechanistic Target of Rapamycin Kinase; *MyD88*, Myeloid Differentiation Primary Response 88; *MYH9*, Myosin Heavy Chain 9; *NCALD*, Neurocalcin Delta; *NOS*, Nitric Oxide Synthase; *NQO1*, NAD(P)H Quinone Dehydrogenase 1; *NPHS1*, NPHS1, Nephrin; *NPY*, Neuropeptide Y; *PACRG*, Parkin Coregulated; *PAI1*, Plasminogen Activator Inhibitor 1; *PARK2*, Parkin RBR E3 Ubiquitin Protein Ligase; *PFKFB2*, 6-Phosphofructo-2-Kinase/Fructose-2,6-Biphosphatase 2; *PLXDC2*, Plexin Domain Containing 2; *PLEKHH2*, Pleckstrin Homology, MyTH4 and FERM Domain Containing H2; *PON*, Paraoxonase; *PPARG*, Peroxisome Proliferators-Activated Receptor Gamma; *PPARGC1A*, Peroxisome Proliferators-Activated Receptor Gamma Co-activator 1 alpha; *PRKAA2*, Protein Kinase AMP-Activated Catalytic Subunit Alpha 2; *PROX1*, Prospero Homeobox 1; *PSMD9*, Proteasome 26S Subunit, Non-ATPase 9; *PRKCB1*, Protein Kinase C Beta; *PTX3*, Pentraxin 3; *PVT1*, Pvt1 Oncogene; *RAGE*, Advanced Glycosylation End-Product Specific Receptor; *RAET1L*, Retinoic Acid Early Transcript 1L; *RBP4*, Retinol Binding Protein 4; REN, Renin; *RGMA*, Repulsive Guidance Molecule BMP Co-Receptor A; *RREB1*, Ras Responsive Element Binding Protein 1; *TOP1MT*, DNA Topoisomerase I Mitochondrial; *RPS12*, Ribosomal Protein S12; *RTN1*, Reticulon 1; SASH1, SAM And SH3 Domain Containing 1; *SCAF8*, SR-Related CTD Associated Factor 8; *SEMA6D*, Semaphorin 6D; *SERPINB*, Serpin Family; *SHROOM3*, Shroom Family Member 3; *SIK1*, Salt Inducible Kinase 1; *SIRT1*, Sirtuin 1; *SLC2A*, Solute Carrier Family 2; *SLC12A3*, Solute Carrier Family 12 Member 3; *SOD*, Superoxide Dismutase; *SOX2*, SRY-Box 2; *SORBS1*, Sorbin and SH3 Domain Containing 1; *SP3*, Sp3 Transcription Factor; *SUMO4*, Small Ubiquitin-Like Modifier 4; *SUV39H2*, Suppressor Of Variegation 3-9 Homolog 2; *TCF7L2*, Transcription Factor 7 Like 2; *TGFβ1*, Transforming Growth Factor Beta 1; *TMPO*, Thymopoietin; *TNFα*, Tumor Necrosis Factor alpha; *THP*, Tamm-Horsfall protein; *TRAF6*, TNF Receptor Associated Factor 6; *TRIB3*, Tribbles Pseudokinase 3; *UMOD*, Uromodulin; *VEGF*, Vascular Endothelial Growth Factor; *VEGFA*, Vascular Endothelial Growth Factor A; *VDR*, Vitamin D Receptor; *WNT4*, Wnt Family Member 4; *ZBTB40*, Zinc Finger and BTB Domain Containing 40; *ZMIZ1*, Zinc Finger MIZ-Type Containing 1.*

The *CNDP1* (carnosine dipeptidase 1) gene is located in chromosome 18q22.3 and contains 5-leucine (CTG) trinucleotide repeat length polymorphism (D18S880) in the coding region (Wanic et al., 2008). This trinucleotide repeat polymorphism is found to have gender specificity and to confer the susceptibility for DKD and ESRD in T2D (Albrecht et al., 2017b). Furthermore, serum carnosinase (CN-1) activity is negatively correlated with time on hemodialysis (Peters et al., 2016). In addition, several SNPs in this gene are also associated with DKD and ESRD (Janssen et al., 2005; Freedman et al., 2007b; McDonough et al., 2009; Alkhalaf et al., 2010; Mooyaart et al., 2010; Ahluwalia et al., 2011b; Chakkera et al., 2011; Kurashige et al., 2013). Interestingly, an experimental study in BTBR ob/ob mice has demonstrated that treatment with carnosine as the target of CNDP1 improves glucose metabolism and albuminuria, suggesting that carnosine may be a novel therapeutic strategy to treat patients with DKD (Albrecht et al., 2017a).

The *ELMO1* (engulfment and cell motility 1) gene is located on chromosome p14.1 and encodes a member of the engulfment and cell motility protein family. The protein interacts with dedicator of cytokinesis proteins and subsequently promotes phagocytosis and cell migration. Increased expression of *ELMO1* and dedicator of cytokinesis 1 may promote glioma cell invasion (Patel et al., 2010). Furthermore, several SNPs in this gene are found to be associated with DKD in both T1D and T2D (Shimazaki et al., 2005, 2006; Craig et al., 2009; Leak et al., 2009; Pezzolesi et al., 2009a; Hanson et al., 2010; Wu et al., 2013; Alberto Ramirez-Garcia et al., 2015; Bodhini et al., 2016; Hathaway et al., 2016; Mehrabzadeh et al., 2016; Sharma et al., 2016). The variants associated with DKD, however, are different in the several populations studied, suggesting the presence of allelic heterogeneity probably resulting from the diverse ancestral genetic backgrounds of the different racial groups.

The *FRMD3* (FERM domain containing 3) gene is located in chromosome 9q21.32. The *FRMD3* gene is expressed in adult brain, fetal skeletal muscle, thymus, ovaries, and podocytes (Ni et al., 2003). Pezzolesi et al. (2009b) have demonstrated that *FRMD3* expression in kidneys of a DKD mouse model is decreased as compared with non-diabetic mice. Genetic polymorphisms in the *FRMD3* gene are associated with DKD and ESRD in T1D and T2D (Freedman et al., 2011; Al-Waheeb et al., 2016). Furthermore, the members of the bone morphogenetic protein (BMP) interact with *FRMD3*, which implies that *FRMD3* may influence the risk of DKD through regulation of the BMP pathway (Martini et al., 2013; Palmer and Freedman, 2013).

The *MMP9* (matrix metallopeptidase 9) gene is located in chromosome 20q13.12. The MMP family members are involved in the breakdown of extracellular matrix (ECM) in physiological processes, such as tissue remodeling, reproduction and embryonic development, while *MMP9* is the ninth member in the family. *MMP9* may play an essential role in local proteolysis of the extracellular matrix and in leukocyte migration. Moreover, MMPs, including *MMP9*, are zinc-dependent endopeptidases and the major proteases in ECM degradation. There are common variants such as rs3918242 (-1562C/T) and microsatellites (CA)n in the promoter region and several SNPs rs481480, rs2032487, rs4281481, rs3752462 and rs3918242 are found to be associated with the susceptibility to DKD (Hirakawa et al., 2003; Nair et al., 2008; Ahluwalia et al., 2009; Freedman et al., 2011; Cooke et al., 2012; Zhang et al., 2015; Feng et al., 2016).

Both *UMOD* (uromodulin) and *SLC12A3* (solute carrier family 12 member 3) genes are located in the same chromosome but in short and long arms, respectively, i.e., 16p12.3 and 16q13. SLC12A3 is also known as thiazide-sensitive sodium-chloride cotransporter in kidney distal convoluted tubules, which is important for electrolyte homeostasis. Mutations in this gene are characterized by hypokalemic alkalosis combined with hypomagnesemia, low urinary calcium, but increased renin activity. Tanaka et al. (2003) performed a GWAS in Japanese T2D subjects and reported that the *SLC12A3* Arg913Gln polymorphism was associated with reduced risk of DKD. Nishiyama et al. (2005) then conducted another 10-year longitudinal study in the same population. The results confirmed that the 913Gln allele of *SLC12A3* Arg913Gln polymorphism conferred a protective effect in DKD (Nishiyama et al., 2005). More recently, Abu Seman et al. (2014) performed a further genetic study of *SLC12A3* polymorphisms in a Malaysian population, including the meta-analysis of the association between the *SLC12A3* Arg913Gln polymorphism and DKD from all the previous studies. *SLC12A3* Arg913Gln polymorphism was found to be associated with T2D (*P* = 0.028, OR = 0.772, 95% CI = 0.612–0.973) and DKD (*P* = 0.038, OR = 0.547, 95% CI = 0.308–0.973) in the Malaysian cohort. The meta-analysis confirmed the protective effects of the *SLC12A3* 913Gln allele in DKD (Z-value = -1.992, *P* = 0.046, OR = 0.792). In addition, the authors investigated the role of *slc12a3* expression in the progress of DKD with db/db mice and in kidney development with zebrafish embryos. With knockdown of zebrafish ortholog, slc12a3 led to structural abnormality of kidney pronephric distal duct at 1-cell stage. Slc12a3 mRNA and protein expression levels were upregulated in kidneys of db/db mice from 6, 12, and 26 weeks at the age. The authors thus concluded that *SLC12A3* is a susceptibility gene in DKD, while allele 913Gln but not allele Arg913 has a preventive effect in the disease (Abu Seman et al., 2014). This association of the *SLC12A3* Arg913Gln polymorphism with DKD has been very recently replicated in a Chinese population (Zhang et al., 2018). The *UMOD* gene encoded glycoprotein is synthesized exclusively in renal tubular cells and released into urine. Furthermore, UMOD may prevent urinary tract infection and inhibit formation of liquid containing supersaturated salts and subsequent formation of salt crystals. SNPs rs4293393 and rs1297707 in the *UMOD* gene are found to be associated with the susceptibility to DKD in T2D (Ahluwalia et al., 2011a; Prudente et al., 2017; van Zuydam et al., 2018).

The Human Genome Project has revealed that there are more than twenty thousand protein coding genes, and probably more than one million of RNA genes^6^. Genetic association studies of RNA gene polymorphisms with DKD are very limited. Up to date, only two SNPs, i.e., rs2910164 and rs12976445 in the genes for miRNA-146a and miRNA-125 have been found to be associated with DKD in T1D and T2D (Li et al., 2014; Kaidonis et al., 2016). Further investigation of RNA genetic variation conferring susceptibility to DKD needs to be undertaken.

## Current Information From Epigenetic Studies in Diabetic Kidney Disease

Similar to genetic association studies, epigenome-wide (EWAS) and candidate gene DNA methylation analyses have been used for epigenetic studies of DKD. Current information from epigenetic studies in DKD are represented in Table 3. An EWAS suggested that several genes, including *SLC22A12, TRPM6, AQP9, HP, AGTX*, and *HYAL2*, may have epigenetic effects in DKD (VanderJagt et al., 2015). Interestingly, *SLC22A12* encodes for urate anion transporter 1 (URAT1), which is a kidney-specific urate transporter that transports urate across the apical membrane of the proximal tubule in kidneys. Loss-of-function *SLC22A12* mutations are associated with renal hypouricaemia and affected persons can develop exercise-induced acute kidney injury and are at increased risk of developing urate stones (Lee et al., 2008). TRPM6 is a member of transient receptor potential superfamily of cation channels. This gene is widely expressed in the body, including kidneys along the nephron. The TRPM6 channels are mainly located in the renal distal convoluted tubule, the site of active transcellular calcium and magnesium transport in the kidney (Felsenfeld et al., 2015). As described previously, several studies have implicated *UMOD* genetic polymorphisms in the susceptibility to DKD (Ahluwalia et al., 2011a; Prudente et al., 2017; van Zuydam et al., 2018). A recent study has demonstrated that UMOD regulates renal magnesium homeostasis through TRPM6 (Nie et al., 2018). Furthermore, analyses of the candidate genes such as *IGFBP1* and *MTHFR* have also provided evidence that DNA methylation changes in these genes may be involved in the pathogenesis of DKD (Gu et al., 2013, 2014; Yang et al., 2016). Combining and analyzing data from genetic and epigenetic studies together may help understand some of the pathophysiology in DKD.

**Table 3:** Current information from epigenetic studies in diabetic kidney disease.

Analysis	Gene symbol/ Target	Material and methods	Results	References
DNA methylation	*AKR1B1, TIMP-2*	T2DM-DKD	Hypomethylation of the genes are associated with albuminuria	Aldemir et al., 2017
	***AKR1B1, IGF1, SLC12A3***	T2DM-DKD and ESRD	Those genes implicated in DKD based upon the inter-individual epigenetic differences	Sapienza et al., 2011
	*CTGF*	T2DM-DKD Glomerular and mesangial cells	Hypomethylation through the decreased Dnmt3a binding in the gene promoter	Zhang et al., 2014
	*IGFBP1*	T1DM-DKD	Hypermethylation	Gu et al., 2014
	*IL13RA1, IL15, EDG3, INHA*	Hemodialyzed patients with DKD	Hypermethylation	Korabecna et al., 2013
	*MTHFR*	Diabetic complications, including DKD	Hypermethylation	Dos Santos Nunes et al., 2018
	*MTHFR*	T2DM-DKD	Demethylation	Yang et al., 2016
	*MIOX*	Human and mouse	Hypomethylation	Sharma et al., 2017
	*PIK3C2B*	Glomeruli in DKD	Up-regulated with methylation in glomeruli	Wang et al., 2018
	*POLR2G, DDB1, ZNF230*		Down-regulated with methylation in glomeruli	
	*SLC30A8*	T2DM-DKD	Hypermethylation	Seman et al., 2015
	***SLC22A12, TRPM6, AQP9, HP, AGXT, HYAL2***	Pre-diabetes and T2DM-DN	Hypermethylation found in 174 of 694 CpG sites	VanderJagt et al., 2015
	***TAMM41, PMPCB, TSFM, AUH***	T1DM-DKD	DNA methylation changes in these genes and influence with mitochondrial function	Swan et al., 2015
	***UNC13B***	T1DM-DKD	An intronic polymorphism rs13293564 in the gene is associated with DKD DNA methylation levels in 19 CpG sites are changed	Bell et al., 2010
	*KLF4*	Glomerular podocytes in human and mouse	DNA methylation levels in the promoters of genes encoding mesenchymal markers are increased	Hayashi et al., 2014
	*aPC*	Podocytes	aPC epigenetically controls p66(Shc) expression	Bock et al., 2013
	*egfr*	Cultured proximal tubule (normal rat kidney) cells	Inhibition of histone deacetylase in eGFR	Gilbert et al., 2011
	*pxr*	db/db mice and proximal tubular cells	Demethylation of DNA	Watanabe et al., 2018
	*dnmt1*	db/db mice	Hypomethylation	Zhang et al., 2017
	*agt, abcc4, cyp4a10, glut5*	db/m mouse	Hypomethylation	Marumo et al., 2015
	*kif20b, cldn18, slco1a1*		Hypomethylation	
	*sglt2, pck1, g6pc, hnf4a*	db/db mice	Demethylated in the proximal tubules	Marumo et al., 2015
	*tgfb1, tet2*	db/db mice	Decreased DNA methylation	Yang et al., 2018
Histone modification	*MTHFR*	T2D with DN	MTHFR regulates histone modification rs1801133 C677T in the gene is associated with DN	Zhou et al., 2015
	*TGFB1*	Glomerular and mesangial cells	TGF-β1 increases expression of the H3K4 methyltransferase SET7/9	Sun et al., 2010
	*12/15-LO*	Glomerular and mesangial cells	Up-regulation of histone lysine modifications	Yuan et al., 2016
	*h3k9/14ac, at1r*	Glomerular and mesangial cells db/db mice	Losartan attenuated increased H3K9/14Ac at RAGE, PAI-1 and MCP-1 promoters, while the chromatin state at these genes are mediated in part by AT1R	Reddy et al., 2014
	*h3k9, h3k23*	db/db and C57BL/6 mice	Acetylation	Sayyed et al., 2010
	*h3k4* in serine 10		Demethylation and phosphorylation	
	*h3k9/14ac*	db/+ mice	Losartan reversed permissive epigenetic changes in renal glomeruli	Reddy et al., 2014
	*set7/9*	db/db mice	Induced histone modification and mcp-1 expression	Chen et al., 2014
	*xbp1*	db/db mice	XBP1s-mediated of histone SET7/9 and consequently decreased MCP-1 expression	Chen et al., 2014
	*opn/h3k27me3*	Sur1-E1506K mice	Histone modification with *opn*	Cai et al., 2016
	*txnip, h3k9ac, h3k4me3, h3k4me1, h3k27me3*	Sur1-E1506K mice	Histone acetylation changes	De Marinis et al., 2016
	*egfr*	Cultured proximal tubule (normal rat kidney) cells	Inhibition of histone deacetylase in eGFR	Gilbert et al., 2011
	*grp78/histone h4*	Diabetic rats	Acetylation changes	Sun et al., 2016
	*mfn2*	Diabetic rats	Histone acetylation at collagen IV promoter	Mi et al., 2016
	h3 and hsp-27, map kinase p28	Sprague-Dawley rats	Dephosphorylation and acetylation of h3	Tikoo et al., 2008
Non-coding RNA dysregulation	miR-9-3, miR34a, miR-137	DKD and diabetic retinopathy	DNA methylation changes	Dos Santos Nunes et al., 2018
	miR-199b-5p, klotho	T2DM-DKD and STZ mice	Increased serum klotho levels are mediated by miR-199b-5p	Kang and Xu, 2016
	microRNA Let-7a-3	T2DM with DKD	DNA methylation levels in the promoter are increased by targeting UHRF1	Peng et al., 2015
	microRNA 1207-5P	Glomerular and mesangial cells	This PVT1-derived microRNA is upregulated by glucose and TGF-β1	Alvarez et al., 2013
	*creb1*, miR-10a	HFD/STZ mice	This microRNA regulate epigenetic modification by targeting creb1	Shan et al., 2016

*DKD, Diabetic Kidney Disease; T1D, Type 1 Diabetes; T2DM, Type 2 Diabetes. The genes predicted by epigenome-wide association analysis are shown in bold, while genes from rodent studies are shown in lower case. *AKR1B1*, Aldo-Keto Reductase family 1, member B1; *aPC*, activated Protein C; *AQP9*, Aquaporin; *AT1R*, Angiotensin II Receptor type 1; *AUH*, AU RNA binding protein/enoyl-CoA hydratase; *EGFR*, epidermal growth factor receptor; *CTGF*, Connective Tissue Growth Factor; *DDB1*, Damage Specific DNA Binding Protein 1; *EDG3*, Endothelial Differentiation G-protein coupled receptor 3; *DNMT1*, DNA methyltransferase 1; *HFD*, High Fat Diet; *IGF1*, Insulin like Growth Factor 1; *IGFBP1*, Insulin-like Growth Factor Binding Protein 1; *IL13RA1*, interleukin 13 receptor subunit alpha 1; *IL15*, Interleukin 15; *INHA*, Inhibin alpha; *KLF4*, Kkruppel-like factor 4; *MTHFR*, Methylenetetrahydrofolate Reductase; *MIOX*, Myo-Inositol Oxygenase; *PIK3C2B*, Phosphatidylinositol-4-Phosphate 3-Kinase Catalytic Subunit Type 2 Beta; *PMPCB*, Peptidase, Mitochondrial Processing beta subunit; *POLR2G*, RNA Polymerase II Subunit G; *SLC12A3*, Solute Carrier family 12 member 3; *SLC22A12*, Solute Carrier family 22 member 12; *SLC30A8*, Solute Carrier family 30 member 8; *TAMM41*, TAM41 Mitochondrial translocator assembly and maintenance homolog; tet2, tet methylcytosine dioxygenase 2; *TIMP2*, TIMP metallopeptidase inhibitor 2; *TRPM6*, Transient Receptor Potential cation channel subfamily M member 6; *TSFM*, Ts translation elongation Factor, Mitochondrial; *UHRF1*, Ubiquitin like with PHD and Ring Finger domains 1; UNC13B, Unc-13 homolog B member 3; *XBP1*, X-Box Binding Protein 1; *ZNF230*, Zinc Finger Protein 230; *12/15-LO*, 12/15-lipoxygenase; *TGFB1*, Transforming Growth Factor Beta 1.*

ncRNAs regulate gene expression at the post-transcriptional level and are involved in chromatin histone modification. Most of studies concerning histone modification and ncRNA dysregulation have been performed in diabetic animal models, while a few studies have been undertaken in subjects with DKD (Table 3). Reddy et al. (2014) have analyzed histone modification profiles in genes associated with DKD pathology and the modified regulation of these genes following treatment with the angiotensin II type 1 receptor (AT1R) blocker losartan. The data indicate that losartan attenuates key parameters of DKD and modifies gene expression, and reverses some epigenetic changes in db/db mice. Losartan also attenuates increased H3K9/14Ac at RAGE, PAI-1, and MCP-1 promoters in mesangial cells cultured under diabetic conditions (Reddy et al., 2014). In a recent study of subjects of T2D and diabetic complications (including DKD) (Dos Santos Nunes et al., 2018) the methylation profiles of miR gene were compared and related to the presence of diabetic complications. Results indicated that miRs can modulate the expression of a variety of genes and methylation changes of *miR*-*9*-*3, miR*-*34a*, and *miR*-*137* were found to be associated with diabetic complications (Dos Santos Nunes et al., 2018). These two studies provide evidence suggesting that therapies targeting epigenetic regulators might be beneficial in the treatment of DKD.

## Summary and Perspectives

Researchers have made major efforts to undertake well powered genetic and epigenetic studies in DKD to help understand its pathogenesis. The data, however, need to be confirmed by several strategies, for instance, replication studies could be performed with better selection of subjects with similar genetic background to limit influences from migration; intermarriage; cultural preferences; coupled with further investigation of DNA variation and methylation changes in RNA regulation genes and biological experiments to determine functional impact of these variants. Furthermore, new technologies for DNA and ncRNA sequencing analysis such as third generation sequencing and a PheWAS approach have recently been developed.

### New Generation Sequencing

DNA sequencing analysis is used for determining the accurate order of nucleotides along chromosomes and genomes. Second-generation sequencing, commonly known as next-generation sequencing (NGS), has presently become popular in DNA sequencing analysis because NGS can enable a massively-paralleled approach capable of producing large numbers of reads at high coverages along the genome and therefore dramatically reduce the cost of DNA sequencing analysis (Treangen and Salzberg, 2011; Gu et al., 2018; Mone et al., 2018). Today, third-generation sequencing (often called as long-read sequencing) is a new generation sequencing method, which works by reading the nucleotide sequences at single molecule level in contrast to the first and second generations of DNA sequencing (van Dijk et al., 2018). Moreover, it is necessary to develop the molecular instruments for whole genome sequencing to make this new generation sequencing commercially available. The advanced sequencing technologies will improve genetic and epigenetic studies in DKD in the near future.

### ncRNA Genetic and Epigenetic Studies

In the human genome, RNA genes are much more abundant than protein coding genes, while ncRNAs mainly include miRNAs and lncRNAs. Both forms of ncRNAs have been found to be involved in chromatin histone modifications, and subsequently can have epigenetic effects on the target genes. Therefore, identification of RNA genetic variation and investigation of biological alteration of these RNA genes should be included in research plans. Kato has very recently pointed out a hypothesis that transforming growth factor-β (TGF1β) may play an important role in early stage development of DKD, while some miRNAs and lncRNAs regulate the key molecules in the TGF1β pathway. These ncRNAs may be served as biomarkers for predicting the potential targets for prevention and treatment in DKD (Kato, 2018). Furthermore, Smyth et al. (2018) have compared Sanger sequencing and NGS to validate the five top ranked miRNAs that are predicted to be associated with DKD by EWAS. This study suggests that targeted NGS may offer a more cost-effective and sensitive approach and implied that the methylated miR-329-2, in which region SNP rs10132943 is located, and miR-429 where SNPs rs7521584 and rs112695918 exist, are associated with DKD (Smyth et al., 2018). Although these two studies are preliminary, they may be good examples to help direct further DKD research.

### Phenome-Wide Association Study (PheWAS)

PheWAS is a new approach to analyze many phenotypes in comparison with a single genetic variant. This approach was originally described using electronic medical record (EMR) data from EMR-linked with a DNA biobank and also can be combined with GWAS and EWAS. Therefore, PheWAS has become a powerful tool to investigate the impact of genetic variation on drug response among many individuals and may expand our knowledge of new drug targets and effects (Pendergrass and Ritchie, 2015; Denny et al., 2016; Roden, 2017). Clearly, combined with GWAS and EWAS, PheWAS will provide us with the possibility to discover the associations with drug effects, including therapeutic response and side effect profiles in DKD (Hebbring, 2014).

Taken together, application of these advanced studies in DKD will be very useful not only for evaluating current data from genetic and epigenetic studies but also for generating new knowledge for dissecting the complexity of this disease.

## Author Contributions

The author confirms being the sole contributor of this work and has approved it for publication.

## Conflict of Interest Statement

The author declares that the research was conducted in the absence of any commercial or financial relationships that could be construed as a potential conflict of interest.

## Footnotes

1 http://www.diabetesatlas.org/

2 https://www.ncbi.nlm.nih.gov/feed/rss.cgi?ChanKey=dbsnpnews

3 https://genome.ucsc.edu/cgi-bin/hgTables

4 https://en.wikipedia.org/wiki/CpG_site

5 http://karokidney.org

6 https://www.genecards.org/

## Abbreviations

ACR: albumin-to-creatinine ratio
ADA: American Diabetes Association
BMI: body mass index
CNV: copy number variant
DKD: diabetic kidney disease
ESRD: end-stage renal disease
EWAS: epigenome-wide association study
GFR: glomerular filtration rate
GWAS: genome-wide association study
IDF: International Diabetes Federation
IHME: Institute for Health Metrics and Evaluation
LD: Linkage disequilibrium
PheWAS: phenome-wide association study
SNP: single nucleotide polymorphism
T1D: type 1 diabetes
T2D: type 2 diabetes
UAE: urinary albumin excretion
